# Supplementary material for: Long-period microseismicity reveals cryptic earthquake-triggered fluid activity can facilitate caldera eruptions
Source: Nat Commun. 2026 Jan 26;17:2040. doi: 10.1038/s41467-026-68645-4 (PMC12946353; doi:10.1038/s41467-026-68645-4)
Supplement: Supplementary file 1 — Supplementary Information [file 41467_2026_68645_MOESM1_ESM.pdf]

Long-period microseismicity reveals cryptic earthquake-triggered fluid activity can facilitate caldera eruptions

Author Information

Zilin Song[1, 2], Andrew F. Bell[2, *], Peter C. LaFemina[3, 4], Sophie Butcher[2, 5], Mario Ruiz[6], Stephen Hernández[6], Patricia M. Gregg[7], Yen Joe Tan[1,*]

Affiliations

[1]Department of Earth and Environmental Sciences, The Chinese University of Hong Kong, Hong Kong S.A.R., China.

[2]School of Geosciences, University of Edinburgh, Edinburgh, UK.

[3]Alfred Wegener Institute, Helmholtz Center for Polar & Marine Research, Bremerhaven, Germany.

[4]Faculty of Geosciences, University of Bremen, Bremen, Germany.

[5]British Geological Survey, Edinburgh, UK.

[6]Instituto Geofísico, Escuela Politécnica Nacional, Quito, Ecuador.

[7]Department of Earth Science & Environmental Change, University of Illinois at Urbana–Champaign, Champaign, IL, USA.

Contents of this file

Supplementary Methods

Supplementary Figures 1-9

Supplementary References

Supplementary Methods

1. Machine-learning based Event Catalogue

We begin with Volpick, an EQTransformer-based phase picker for volcano seismicity developed by Zhong and Tan (2024), to pick P- and S-arrival times for VTs and LPs across seismic stations on 26 June 2018. All seismograms are first resampled to 100 Hz, bandpass filtered between 2-12 Hz, detrended, and normalized. Continuous waveforms are segmented using 60-second time windows as input to the deep-learning phase picker, with a 50 % overlap between consecutive segments maintained (Tan et al., 2021). With a probability threshold of 0.2 (Mousavi et al., 2020), 13144 P- and 12196 S- picks are identified.

Phase arrivals are associated into individual earthquakes through PyOcto (Münchmeyer, 2024), with event locations further refined by grid-search inversion. The search area is centered within the caldera, spanning 0.14˚ horizontally and extending 30 km depth, with a grid spacing of 1 km. We use the 1-D velocity model from Rezaeifar et al. (2024). Earthquakes are declared when a minimum of 4 P picks and 1 S pick are associated with a residual tolerance of 0.5 s. Events occurring within 2 s time intervals are identified as duplicates, those with fewer assigned picks will be systematically eliminated (Zhou et al., 2021). As a result, 994 events are successfully derived and located from 6261 P and 5159 S arrivals. Hypocentral locations are further refined using HypoInverse software (Klein, 2002), resulting in mean absolute location errors of 0.8 km and 2.8 km, along with median values of 0.5 km and 0.8 km in horizontal and vertical dimensions, respectively. The average RMS residual is calculated to be 0.13 s. We then select 785 earthquakes with ideal station coverage (azimuth gaps < 300˚) and location errors (< 5 km) for relocation using HypoDD double-difference method (Waldhauser & Ellsworth, 2000). Event pairs located within 5 km from each other are used for differential times calculations. We determine phase-derived time differences for individual event pairs with at least 4 observations at common stations. Cross-correlation-derived differential times are calculated using 1.5 s time windows, beginning 0.5 s

before P picks on the vertical channel, and 3 s time windows, starting 1 s before S picks on the horizontal channel, for seismic signals with signal-to-noise ratios (SNR) above 2. Time shifts are only considered when the normalized cross correlation value (NCC) exceeds 0.7 (Power et al., 2021; Rodgers et al., 2016; Shakirova & Chemarev, 2023). Eventually, a total of 694 earthquakes is successfully relocated for further analysis. Through an iterative process of randomly selecting and relocating earthquakes from the relocated earthquake catalogue using singular value decomposition mode (Waldhauser & Ellsworth, 2000), average location uncertainties are determined to be 0.3 km and 0.4 km in horizontal and vertical dimensions, respectively.

Local magnitude (M_L) for earthquakes that are estimated following Bakun and Joyner (1984):

$$M_L = \log_{10}(A_{Peak}) + \log_{10}(R/100) + 0.00301*(R-100) + 3.0 \quad (1)$$

Where A_{Peak} represents the maximum amplitude of horizontal ground motion at the epicentral distance (R). After deconvolving instrument response from raw waveforms and convolving with the theoretical Wood-Anderson seismometer response (Zhang et al., 2019), we use a 5 s time window, beginning 1s before S arrivals, to measure the average peak-to-peak amplitude (A_{Peak}) at the horizontal components of each station. The M_L for each earthquake is determined by the median measurements across the station network.

2. Enhanced Catalogue via Template Matching

We apply a template matching algorithm through EQcorrscan (Chamberlain et al., 2017), a cross-correlation-based earthquake detection Python package, to further enhance our catalogue. First, waveforms are pre-processed by sampling at 50 Hz and filtering within 2-20 Hz to minimize memory cost and ensure manageable calculation workload. Based on the machine-learning based event catalogue, template events are selected if their P picks on the vertical channel and S picks on the horizontal channel are well recorded (SNR $\geqslant$ 2) by at least 4 stations. Consequently, 669 templates are determined and cross

correlated with continuous data recorded on 26 June 2018. Template waveforms are constructed from 5 s time windows starting 1 s before seismic phase arrivals, with P-arrivals from vertical channels and S-arrivals from horizontal channels. To ensure a robust detection process, we cut the continuous data into hourly chunks with a 30-second overlap between consecutive segments and remove chunks with excessive data gaps and spikes before template matching.

Detections are declared when the sum of NCC values across stations exceeds 9 times the median absolute deviation (Shelly et al., 2007). This conservative threshold can re-discover 99 % of the templates from the continuous data. We consider detections with an average NCC across stations below 0.4 as false detections and remove them from the analysis. Detections with origin time differences of less than 2 s from templates are removed as duplicates. For detections with an interevent times less than 2 s, we keep the one with the highest NCC value (van Wijk et al., 2021). Ultimately, 1241 earthquakes are newly catalogued and assumed to be co-located with their template events (Fig. 2b, 4a-d).

We estimate M_L for template-matching detections ($M_{Detection}$) based on magnitudes of the associated templates ($M_{Template}$) following:

$$M_{Detection} = M_{Template} + c * \log(\alpha) \quad (2)$$

Where c, a scaling constant estimated to be 0.7 (Schaff, 2008; Shelly et al., 2016; Fig. S6), is employed for correcting amplitude ratios (α) between detections and associated templates. We measure α at different stations by performing principal component analysis on 5 s pre-processed waveforms, starting 1 s before P arrivals at the vertical channel. For each detection, median α over stations is taken as the final estimate.

Supplementary Figures

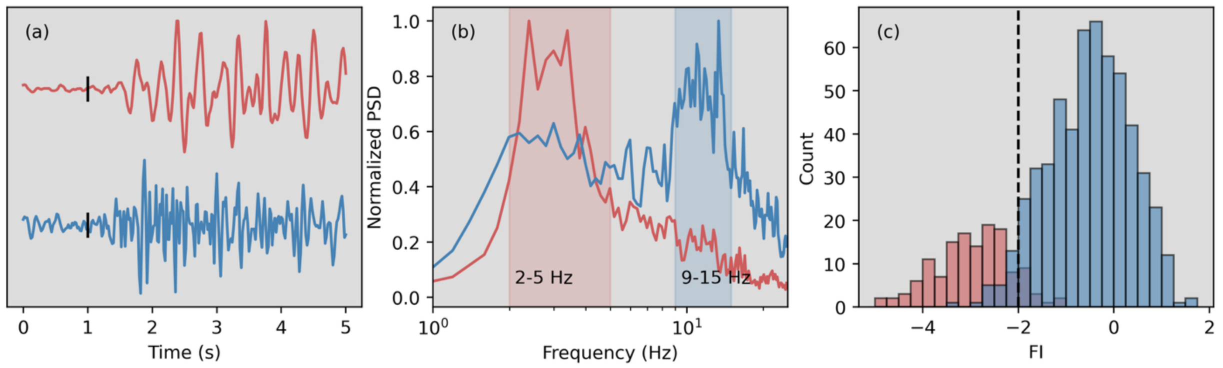

Supplementary Figure 1. (a) Sample waveforms of a calibration long-period earthquake (LP; red) and volcano-tectonic earthquake (VT; blue) recorded by the vertical channel of station SN05. Waveforms are filtered within the 2-20 Hz frequency band. To enhance microseismicity classification, we selected 5 largest-magnitude manually-labeled LPs and 5 largest-magnitude manually-labeled VTs (all magnitudes < 3) as calibration events. (b) Stacked spectra of 5 manually classified calibration LPs (red) and VTs (blue). Spectra for each event are calculated across stations with high-quality recordings (signal-to-noise ratio ≥ 2). The low and high frequency bands applied for frequency index (FI) calculation are highlighted by red and blue shaded regions, respectively. (c) Histograms of FI values for manually classified earthquakes, with red and blue colors representing LPs and VTs, respectively. The vertical dashed line indicates local minimum at FI = -2, defining the threshold for systematic earthquake classification.

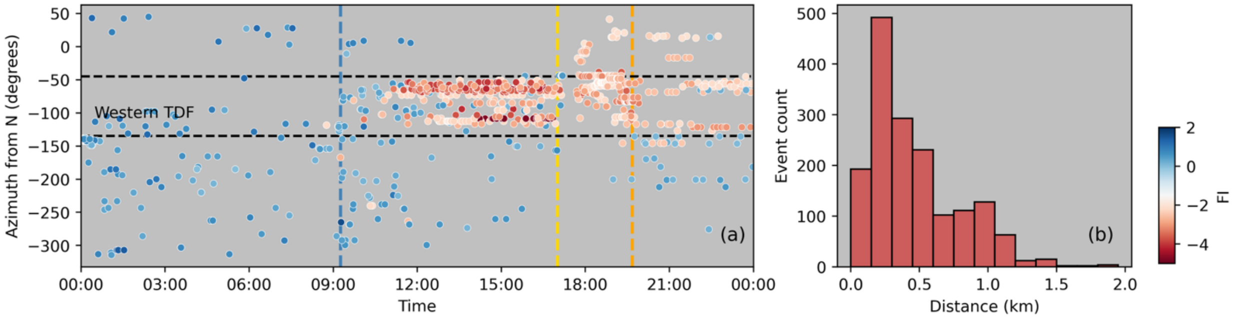

Supplementary Figure 2. (a) Spatiotemporal distributions of LPs (red dots), identified using FI, and typical VTs (blue dots). VTs with FI > 0 are selected as typical events. (b) Histograms of distances between LPs and the closest typical VTs.

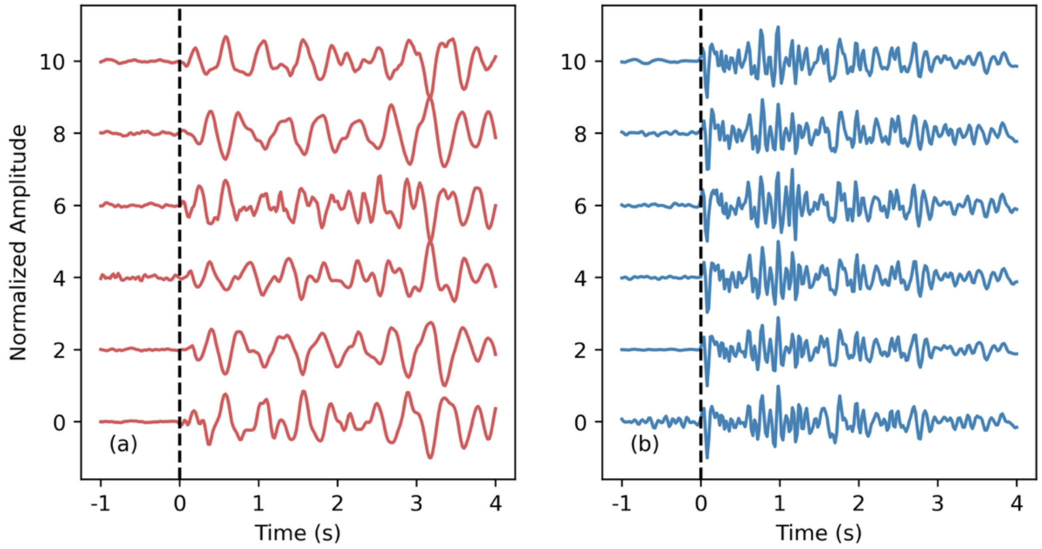

Supplementary Figure 3. Sample waveforms of LP (a) and VT (b) multiplets. Waveforms are recorded by the vertical channel at station SN12 and filtered within the frequency band of 2-20 Hz. Waveforms have been aligned using time shifts determined from maximum cross-correlation values, with dashed lines indicating the P arrivals of the bottom waveforms as the reference time.

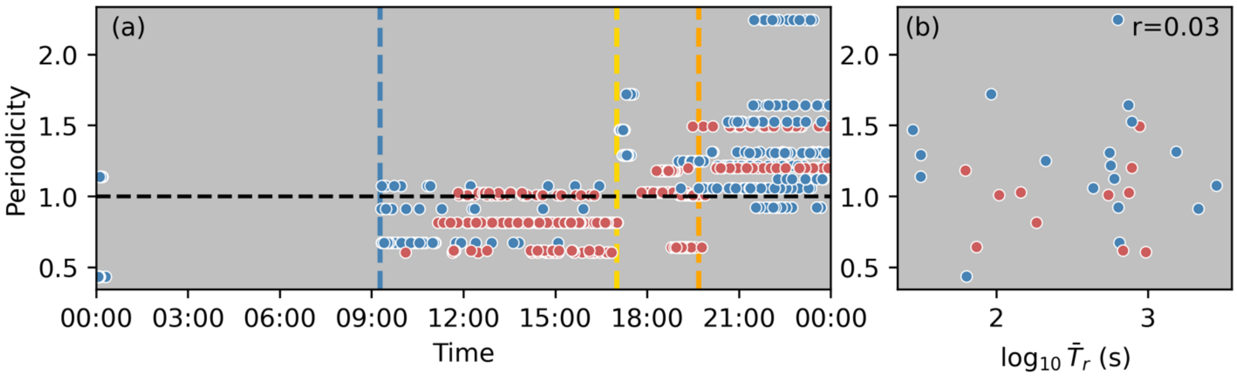

Supplementary Figure 4. (a) Periodicity distribution of multiplet families, with LP (red) and VT (blue) families classified based on the average FI value of multiplets within each family, using a threshold of -2. Horizontal dashed line represents periodicity threshold of 1 while vertical dashed lines indicate onset times of the moment magnitude (M_w) 5.4 earthquake, magma intrusion, and eruption as presented in Figure 2. (b) Pearson correlations (r) between the average recurrence time ($\overline{T_r}$) and periodicity for multiplet families.

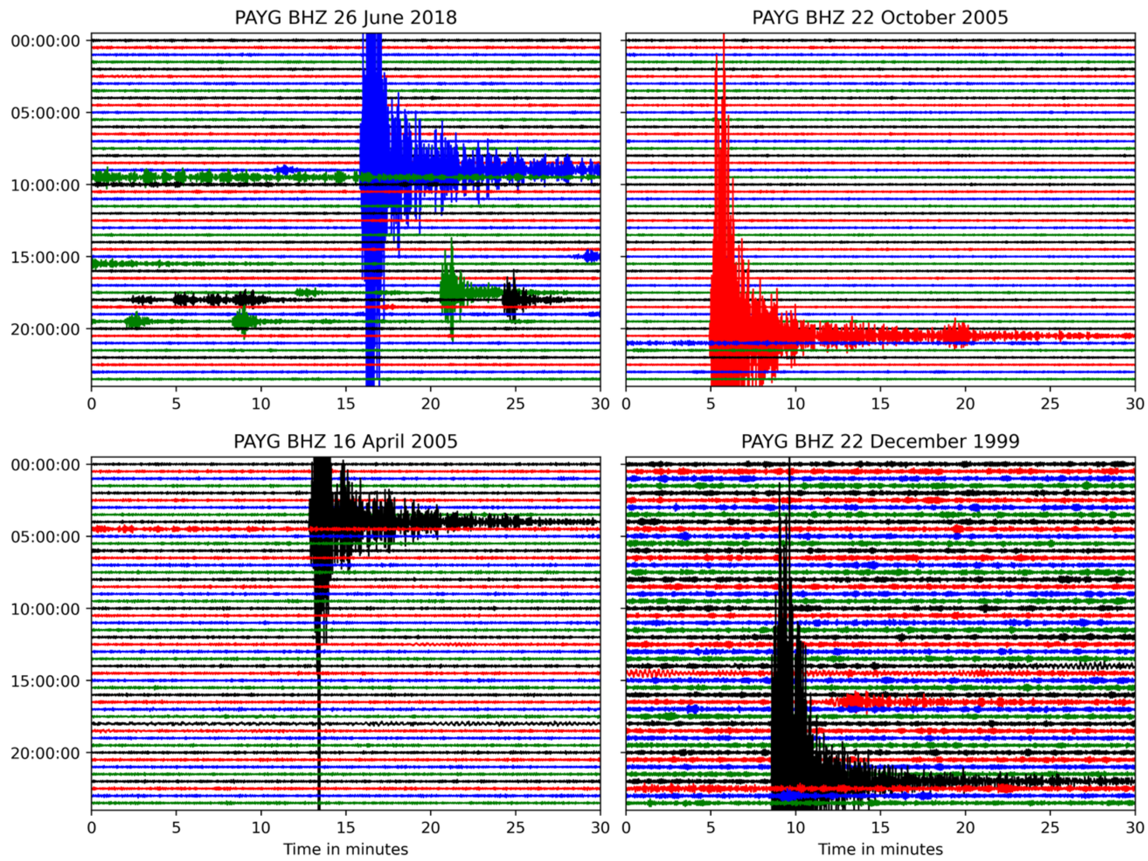

Supplementary Figure 5. Day plots of true ground motion seismic velocity from the vertical component of the PAYG seismometer, with UTC timestamps on the y-axis.

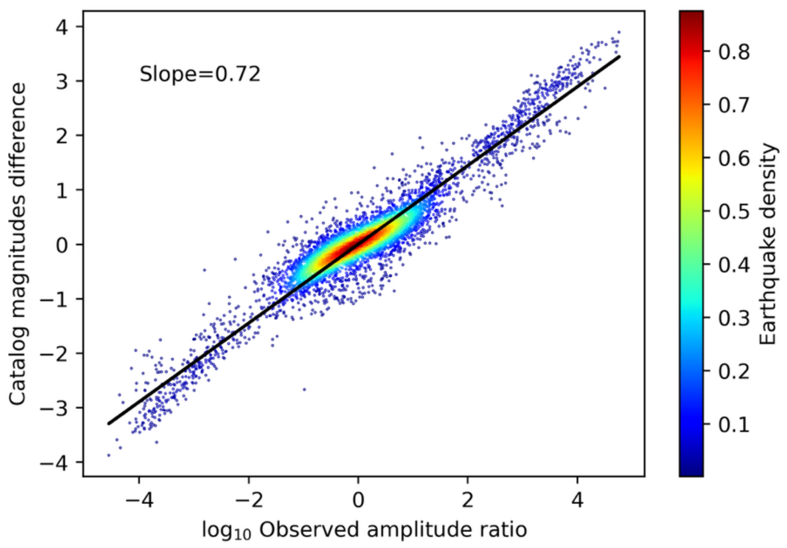

Supplementary Figure 6. Scaling between observed amplitude ratios and catalog magnitude differences for template earthquakes. The black line indicates the principal component fit for data samples.

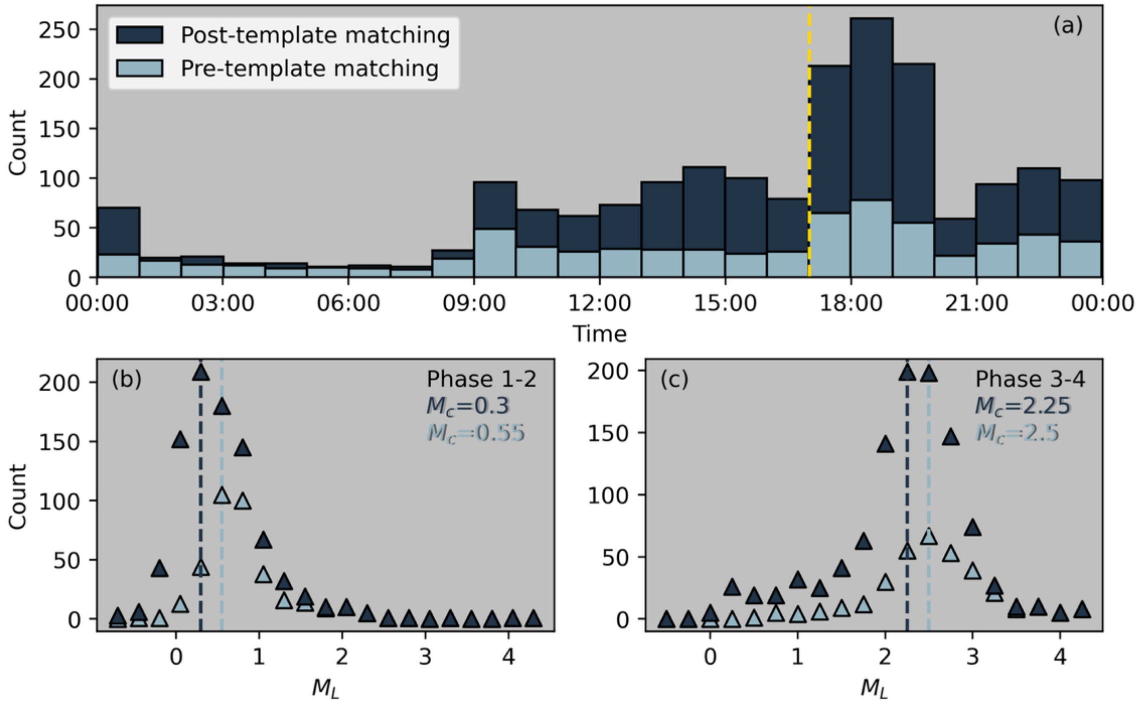

Supplementary Figure 7. (a) Comparison of earthquake catalogues before and after applying template matching techniques. The yellow dashed line denotes the onset time (17:00) of magma intrusion (Bell et al., 2021b). (b-c) Magnitude frequency distributions of events during Phases 1-2 (b) and 3-4 (c) for pre- (light blue triangles) and post-template-matching (dark blue triangles) catalogues. Dashed lines represent magnitude completeness (M_c) for each earthquake catalogue, determined by max-curvature method (Wiemer & Wyss, 2000).

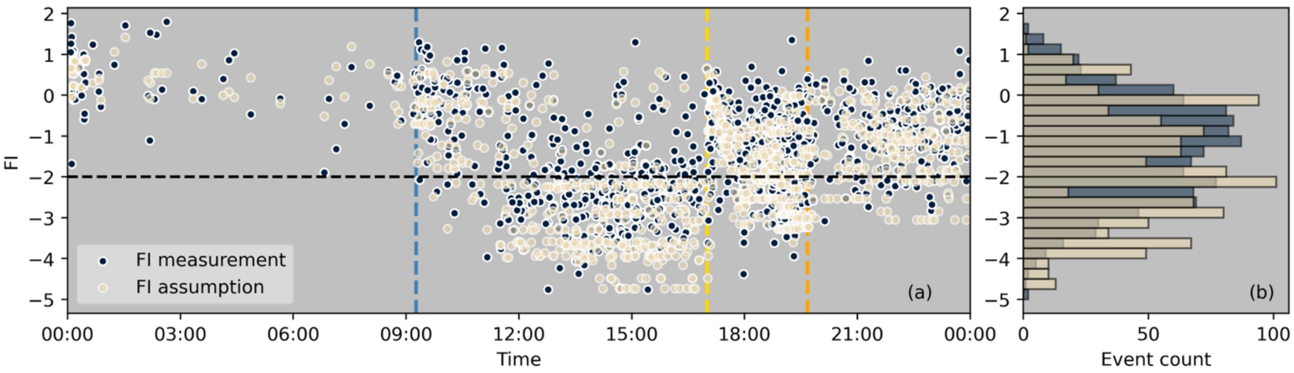

Supplementary Figure 8. (a) Temporal distribution of FI values for template-matching detections derived from template equivalence assumptions (yellow) and real measurements (blue). The horizontal dashed line denotes FI threshold for classifying earthquakes into LP and VT. Vertical dashed lines mark onset times of the M_w 5.4 earthquake, magma intrusion, and eruption, as shown in Figure 2. (b) Histograms of FI values from template equivalence assumptions (yellow) and real measurements (blue).

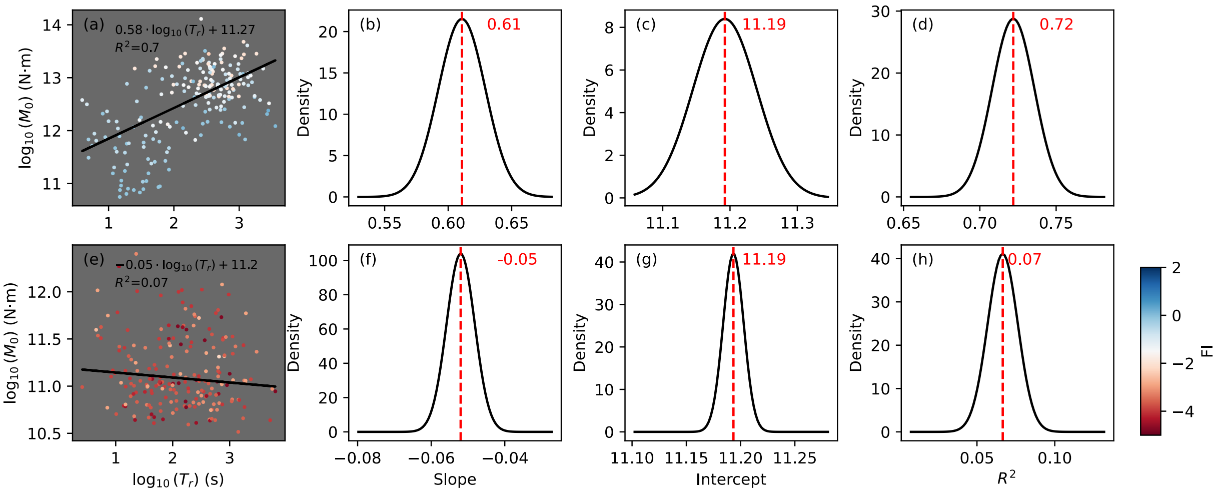

Supplementary Figure 9. Seismic moment (M_0) versus recurrence time (T_r) scaling for post-intrusion VT (a) and pre-intrusion LP (e) multiplet families. Seismic events are represented by circles, color-coded by their FI values. Corresponding linear regressions (black lines; detailed in the text in panels a and e) were calculated using Random Sample Consensus (RANSAC). R^2 indicates the coefficient of determination for each regression. Jackknife resampling was performed on M_0-T_r scaling within these VT (b-d) and LP (f-h) multiplet families. Linear model parameters are fitted to normal distributions, with peaks marked by red dashed lines.

Supplementary References

1. Bakun, W. H., & Joyner, W. B. (1984). The M_L scale in central California. *Bulletin of the Seismological Society of America*, **74**(5), 1827-1843.
2. Chamberlain, C. J., Hopp, C. J., Boese, C. M., Warren-Smith, E., Chambers, D., Chu, S. X., Michailos, K., & Townend, J. (2017). EQcorrscan: Repeating and Near-Repeating Earthquake Detection and Analysis in Python. *Seismological Research Letters*, **89**(1), 173-181, doi: 10.1785/0220170151.
3. Klein, F. W. (2002). User's guide to HYPOINVERSE-2000, a Fortran program to solve for earthquake locations and magnitudes (2331-1258).

4. Mousavi, S. M., Ellsworth, W. L., Zhu, W., Chuang, L. Y., & Beroza, G. C. (2020). Earthquake transformer—an attentive deep-learning model for simultaneous earthquake detection and phase picking. *Nature Communications*, **11**(1), 3952, doi: 10.1038/s41467-020-17591-w.
5. Münchmeyer, J. (2024). PyOcto: A high-throughput seismic phase associator. *Seismica*, **3**(1), doi: 10.26443/seismica.v3i1.1130.
6. Power, J. A., Roman, D. C., Lyons, J. J., Haney, M. M., Rasmussen, D. J., Plank, T., Nicolaysen, K. P., Izbekov, P., Werner, C., & Kaufman, A. M. (2021). Volcanic seismicity beneath Chuginadak Island, Alaska (Cleveland and Tana volcanoes): Implications for magma dynamics and eruption forecasting. *Journal of Volcanology and Geothermal Research*, **412**, 107182, doi: 10.1016/j.jvolgeores.2021.107182.
7. Rezaeifar, M., Bean, C. J., Grannell, J., Möllhoff, M., & Bell, A. F. (2024). Intrusive mechanism of the 2018 Sierra Negra Galápagos eruption, constrained by 4D tomographic images. *Journal of Volcanology and Geothermal Research*, **451**, 108112 doi: 10.1016/j.jvolgeores.2024.108112.
8. Rodgers, M., Smith, P. J., Mather, T. A., & Pyle, D. M. (2016). Quiescent-explosive transitions during dome-forming volcanic eruptions: Using seismicity to probe the volcanic processes leading to the 29 July 2008 vulcanian explosion of Soufrière Hills Volcano, Montserrat. *Journal of Geophysical Research: Solid Earth*, **121**(12), 8453-8471, doi: 10.1002/2016JB013180.
9. Schaff, D. P. (2008). Semiempirical statistics of correlation-detector performance. *Bulletin of the Seismological Society of America*, **98**(3), 1495-1507.
10. Shakirova, A., & Chemarev, A. (2023). Multiplets of low-frequency earthquakes during the eruption of the Kizimen volcano in 2011–2012, Russia. *Journal of Volcanology and Geothermal Research*, **438**, 107805, doi: 10.1016/j.jvolgeores.2023.107805.
11. Shelly, D., Beroza, G. & Ide, S. (2007). Non-volcanic tremor and low-frequency earthquake swarms. *Nature,* **446**, 305–307, doi: 10.1038/nature05666.

12. Shelly, D. R., Ellsworth, W. L., & Hill, D. P. (2016). Fluid-faulting evolution in high definition: Connecting fault structure and frequency-magnitude variations during the 2014 Long Valley Caldera, California, earthquake swarm. *Journal of Geophysical Research: Solid Earth*, **121**(3), 1776-1795, doi: 10.1002/2015JB012719.
13. Tan, Y. J., Waldhauser, F., Ellsworth, W. L., Zhang, M., Zhu, W., Michele, M., Chiaraluce, L., Beroza, G. C., & Segou, M. (2021). Machine-Learning-Based High-Resolution Earthquake Catalog Reveals How Complex Fault Structures Were Activated during the 2016–2017 Central Italy Sequence. *The Seismic Record*, **1**(1), 11-19, doi: 10.1785/0320210001.
14. van Wijk, K., Chamberlain, C. J., Lecocq, T., & Van Noten, K. (2021). Seismic monitoring of the Auckland Volcanic Field during New Zealand's COVID-19 lockdown. *Solid Earth*, **12**(2), 363-373, doi: 10.5194/se-12-363-2021.
15. Waldhauser, F., & Ellsworth, W. L. (2000). A Double-difference Earthquake location algorithm: Method and application to the Northern Hayward Fault, California. *Bulletin of the Seismological Society of America*, **90**(6), 1353-1368, doi: 10.1785/0120000006.
16. Zhang, M., Ellsworth, W. L., & Beroza, G. C. (2019). Rapid Earthquake Association and Location. *Seismological Research Letters*, **90**(6), 2276-2284, doi: 10.1785/0220190052.
17. Zhong, Y., & Tan, Y. J. (2024). Deep-Learning-Based Phase Picking for Volcano-Tectonic and Long-Period Earthquakes. *Geophysical Research Letters*, **51**(12), e2024GL108438, doi: 10.1029/2024GL108438.
18. Zhou, P., Ellsworth, W. L., Yang, H., Tan, Y. J., Beroza, G. C., Sheng, M., & Chu, R. (2021). Machine-learning-facilitated earthquake and anthropogenic source detections near the Weiyuan Shale Gas Blocks, Sichuan, China. *Earth and Planetary Physics*, **5**(6), eepp2021053, doi: 10.26464/epp2021053.
